# Changing Clinical Meaning of Resection Margin Status According to the Treatment Paradigm and the Potential Role of Perioperative Radiotherapy for Patients with Pancreatic Ductal Adenocarcinoma: An Updated Multicenter Retrospective Cohort Study

**DOI:** 10.1245/s10434-025-17389-4

**Published:** 2025-04-30

**Authors:** Won-Gun Yun, Wooil Kwon, Hee Ju Sohn, Youngmin Han, Yoon Soo Chae, Hye-Sol Jung, Young Jae Cho, Chang-Sup Lim, Yoo-Seok Yoon, Jin-Young Jang

**Affiliations:** 1 Department of Surgery and Cancer Research Institute, Seoul National University College of Medicine, Seoul, Republic of Korea; 2 Department of Surgery, Seoul Metropolitan Government-Seoul National University Boramae Medical Center, Seoul National University College of Medicine, Seoul, Republic of Korea; 3 Department of Surgery, Chung-Ang University Gwangmyeong Hospital, Chung-Ang University College of Medicine, Gwangmyeong, Republic of Korea; 4 Department of Surgery, Seoul National University Bundang Hospital, Seoul National University College of Medicine, Seongnam-si, Gyeonggi-do Republic of Korea

## Abstract

**Background:**

Surgeons have focused on obtaining microscopically negative margins and developing perioperative treatment strategies for pancreatic head cancer. However, the clinical significance of resection margin and radiotherapy remains unclear, particularly in neoadjuvant chemotherapy (NAC) settings. Therefore, this study aimed to evaluate the prognostic impact of margin status and perioperative radiotherapy.

**Methods:**

Between 2014 and 2019, the study enrolled 307 patients with pancreatic head cancer who underwent upfront pancreaticoduodenectomy (PD) and 97 patients who underwent NAC followed by PD at three tertiary referral hospitals. The margin status was divided into a three-tier system as follows: R0-wide (tumor-free margin, ≥ 1 mm), R0-narrow (0 mm < margin < 1 mm), and R1 (margin = 0 mm).

**Results:**

In the upfront surgery setting, the groups were arranged in descending order of the 5-year overall survival (OS) rates as follows: R0-wide (39.1%), R0-narrow (25.6%), and R1 (12.5%). In the NAC setting, the groups also could be arranged in descending order of 5-year OS rates as follows: R0-wide (52.2%), R0-narrow (45.5%), and R1 (8.3%). However, the differences in OS between the R0-wide and R0-narrow groups did not reach statistical significance (*P* = 0.587), in contrast to the upfront surgery setting. In the multivariate analyses, concurrent chemo-radiotherapy after surgery was significantly associated with a decreased risk of locoregional recurrence in both treatment settings.

**Conclusions:**

Obtaining a wide margin could enhance prognosis in upfront surgery settings, and obtaining only a narrow margin could be appropriate in NAC settings. In addition, adjuvant radiotherapy could be considered, particularly for patients with margin involvement.

**Supplementary Information:**

The online version contains supplementary material available at 10.1245/s10434-025-17389-4.

The past few decades have witnessed major clinical development in the management of pancreatic ductal adenocarcinoma (PDAC).^1^ Although improvements in survival have been attributed primarily to the development of chemotherapy regimens, surgical resection remains the only treatment option with curative potential.^2–5^ Accordingly, many surgeons have been trying for a long time to select patients who could obtain a microscopically negative margin and to explore the prognostic power of resection margin status.

Several studies have reported widely varying negative resection margin rates ranging from less than 20% to more than 80% for patients who underwent pancreaticoduodenectomy (PD).^6–13^ This range is due to the lack of internationally agreed upon methods of PD specimen-handling and definitions of resection margins.

The 1 mm rule, which means the wide resection margin with R0 of 1 mm or more from tumor cells to the margin, was first proposed by the Royal College of Pathologists in the early 2000s.^14^ Although the International Study Group of Pancreatic Surgery and the eighth edition of the American Joint Committee on Cancer Staging Manual both endorsed the 1-mm rule, guidelines in North America and Japan classically define microscopic residual tumor as the presence of tumor cells at the surface of the resection margin (0 mm rule).^15,16^ Which rule is more predictive of a patient’s outcome has been hotly debated, and many studies about the impact of resection margin involvement on prognosis have showed inconsistent results.^17–20^

Another important factor to consider when evaluating the margin status in PDAC is the increasing use of neoadjuvant chemotherapy (NAC). The indications for NAC are gradually expanding to increase the likelihood of negative resection margins by downstaging and treating early micro-metastasis.^21–24^ However, most studies on the prognostic power of resection margin status have focused on patients who underwent upfront surgery. Whether surgical specimens from patients who have previously received systemic treatment can be evaluated using the same criteria as those used for treatment-naïve surgical specimens remains unclear.

In NAC settings, Maeda et al.^25^ reported that the presence of tumor cells directly at the margin was an independent prognostic factor. However, Schmocker et al.^26^ reported that margin involvement did not affect prognosis. Additionally, in this era of the proven benefit of perioperative chemotherapy, the benefit of radiotherapy when added to chemotherapy remains less understood.^27^

Because the goal of radiotherapy is to avoid locoregional failure, radiotherapy should be assessed in conjunction with resection margin status. Therefore, we aimed to evaluate the impact of margin status on the prognosis for patients who underwent upfront PD and NAC followed by PD and the potential role of perioperative radiotherapy in preventing locoregional failure.

## Methods

### Patient Cohort

This multi-center, retrospective cohort study was approved by the institutional review boards (H-2007-025-1139, B-2201-732-105, and 30-2020-317). This study was conducted in accordance with the 1975 Declaration of Helsinki and its later versions. This work was registered at the Research Registry. This work has been reported in line with the Strengthening the Reporting of Cohort, Cross-Sectional and Case–Control Studies in Surgery (STROCSS) criteria.^28^

Between 2014 and 2019, the study enrolled 451 patients who underwent PD for primary PDAC to allow for sufficient follow-up data at the time of analysis. An extensive medical record review was undertaken to gather clinically relevant treatment, disease, and outcome variables, and to confirm pathologic information. The study excluded patients who had a histology other than adenocarcinoma (*n* = 14), insufficient clinical information (*n* = 14), distant metastasis at the time of diagnosis (*n* = 10), other concomitant primary malignancies (*n* = 4), or pathologic complete response after NAC (*n* = 4), or underwent surgery with macroscopically positive margins (*n* = 1). The study also excluded patients who underwent pathologic complete response because the study focused on surgical margins rather than tumor biology.

Subsequently, 404 qualifying patients were stratified according to the treatment strategy (upfront surgery vs NAC) and resection margin status (Fig. S1). The decision whether to undergo surgery or chemotherapy first was made after a multidisciplinary discussion considering resectability first, but there may have been differences in determining treatment strategy depending on the time.

### Pathologic Assessment and Margin Classification

All resected PD specimens were prepared using a fully standardized approach based on 3- to 5-mm-thickness serial slicing in the axial plane perpendicular to the duodenal axis. Experienced gastrointestinal pathologists evaluated the specimens in compliance with the standard methods involving multicolor inking described by the College of American Pathologists guidelines.^16^ Briefly, the pancreatic neck, bile duct, enteric margin means that proximal duodenal or gastric and distal jejunal margin in pancreaticoduodenectomy, superior mesenteric artery (SMA) and vein (SMV) groove margins, and the circumferential anterior and posterior surfaces were evaluated.

Margin status was classified as R0 or R1 based on the 0 mm rule, which indicates the direct invasion of tumor cells. However, the tumor-free margin, which is the distance from the individual surgical margins to the tumor cells, was reported in millimeters for each margin and circumferential surface. Therefore, we distinguished cases with a tumor-free margin 1 mm or larger (R0-wide), cases with a tumor-free margin smaller than 1 mm (R0-narrow) and cases with tumor cells directly at the margin (R1) to evaluate the details of margin status.^29–31^ The tumor stage was determined based on the eighth edition of the American Joint Committee on Cancer Control staging system.^32^

### Data Collection

We retrospectively reviewed the prospectively collected electronic medical records of 404 qualifying patients. Baseline comparisons of patient demographics, treatment, and radiologic and pathologic data were performed among groups stratified by resection margin status. Patient demographics included age, sex, American Society of Anesthesiologists (ASA) physical status classification, and carbohydrate antigen (CA) 19-9 at the time of diagnosis. Radiologic variables included resectability status at the time of diagnosis according to the 2023 National Comprehensive Cancer Network (NCCN) guideline.^33^ Postoperative complications were classified according to the Clavien-Dindo classification and collected for grade 2 or higher.^34^

Information about treatment, such as the use of perioperative chemotherapy or radiotherapy, also were collected. The NCCN principles for chemotherapy were followed, with specifics carried out in accordance with our protocols and previous studies.^33,35,36^ All patients who underwent surgery for pancreatic cancer are advised to receive adjuvant treatment. The final decision on whether to proceed with adjuvant treatment was reached after discussion with patients about adverse effects of adjuvant treatment and their performance status.

In our centers, adjuvant chemotherapy usually is administered for a period of 6 months, and the usual prescription of adjuvant radiotherapy is 50 Gy to the tumor bed and 45 Gy to the regional lymphatics in 25 fractions using a simultaneous integrated boost for the concurrent chemo-radiotherapy. Follow-up data were retrieved to evaluate the prognosis. Patients were generally followed up by computed tomography of the abdomen and pelvis and CA 19-9 every 3 months until 1 year after surgery, every 6 months until 5 years after surgery, and every year after that.

Overall survival (OS) was measured from the date of PD until death or last hospital visit. Overall recurrence was defined as any form of recurrence that first occurred during the follow-up period. Recurrence was defined as radiologic evidence of recurrent disease. Among the recurrences, locoregional recurrence (LRR) was defined as recurrence in the remnant pancreas, the surgical bed, or locoregional nodes after PD. Locoregional recurrence-free survival (LRRFS) was defined as the time from the date of PD to the first LRR or death or the last hospital visit.

### Statistical Analysis

Categorical variables are expressed as numbers with percentages, and continuous variables are expressed as medians with interquartile ranges (IQRs). To compare the clinical characteristics, the chi-square test was used for categorical variables, and Fisher’s exact test was used as a replacement for the chi-square test when the expected frequency of one or more cells was less than five. For continuous variables, the Kruskal-Wallis test was used after confirmation of variables that did not meet normality after the Shapiro-Wilk test.

Multiple comparisons were adjusted according to Benjamini and Hochberg.^37^ Survival analysis was performed using Kaplan-Meier (KM) estimates and compared using the log-rank test. Cox proportional hazards regression models for calculating hazard ratios (HRs) and 95% confidence intervals (CIs), were used to explore the risk factors for LRR. Variables with a *P* value smaller than 0.10, as determined by univariate analysis, were selected for multivariate analysis.

Throughout the study, statistical significance was set at *P* values lower than 0.05, and marginal significance was set at *P* values lower than 0.10. All statistical analyses were performed using the R software, version 4.2.3 (R Foundation for Statistical Computing).

## Results

### Clinical Characteristics

After 47 of the 451 patients had been excluded, the cohort consisted of 404 patients: 216 men (53.5%) and 188 women (46.5%). The baseline characteristics are summarized in Table 1. The median follow-up time was 26 months (IQR, 12–51 months). The median age was 66 years (IQR, 58–72.0 years).

**Table 1:** Baseline characteristics of all pancreatic cancer patients

Variables	Total *n* (%)	Upfront PD *n* (%)	NAC *n* (%)	*P* Value
Number	404	307	97	
Median age: years (IQR)	66.0	67.0	60.0	< 0.001
	(58.0–72.0)	(60.0–74.0)	(56.0–67.0)	
				> 0.99
M:F	216:188	164:143	52:45	
ASA classification				0.338
I/II	351 (86.9)	270 (87.9)	81 (83.5)	
III/IV	53 (13.1)	37 (12.1)	16 (16.5)	
Resectability^a^				< 0.001
RPC	294 (72.8)	270 (88.0)	24 (24.7)	
BR/LAPC	110 (27.2)	37 (12.0)	73 (75.3)	
CA 19-9 (U/mL)^a^				0.020
>150	185 (45.8)	151 (49.2)	34 (35.1)	
≤150	219 (54.2)	156 (50.8)	63 (64.9)	
T stage				0.874
T1/T2	362 (89.6)	276 (89.9)	86 (88.7)	
T3/T4	42 (10.4)	31 (10.1)	11 (11.3)	
N stage				< 0.001
N0	135 (33.4)	85 (27.7)	50 (51.5)	
N1/N2	269 (66.6)	222 (72.3)	47 (48.5)	
R status				0.004
R0-wide	202 (50.0)	139 (45.3)	63 (64.9)	
R0-narrow	128 (31.7)	106 (34.5)	22 (22.7)	
R1	74 (18.3)	62 (20.2)	12 (12.4)	
Complication				0.822
Yes	114 (28.2)	88 (28.7)	26 (26.8)	
No	290 (71.8)	219 (71.3)	71 (73.2)	
Adjuvant treatment				0.006
Yes	336 (83.2)	246 (80.1)	90 (92.8)	
No	68 (16.8)	61 (19.9)	7 (7.2)	

*PD* pancreaticoduodenectomy; *NAC* neoadjuvant chemotherapy; *IQR* interquartile range; *ASA* American Society of Anesthesiologists; *RPC* resectable pancreatic cancer; *BR/LAPC* borderline resectable/locally advanced pancreatic cancer; *CA 19-9* carbohydrate antigen 19-9

^a^Description based on the time of diagnosis

Of the 404 patients, 307 (76%) underwent upfront PD, and 97 (24%) underwent NAC followed by PD. The patients who underwent upfront PD had a higher proportion of patients with resectable pancreatic cancer, high CA 19-9 (>150 U/mL), metastatic lymph nodes, and R1 margin than those who underwent NAC followed by PD.

The baseline characteristics of 307 patients who underwent upfront PD are summarized in Table 2. The median age was 67 years (60–74 years). Of the 307 patients, 164 were men (53.4%), and 143 were women (46.6%). An R0-wide margin was achieved in 139 (45.3%) patients, an R0-narrow margin in 106 (34.5%), patients and an R1 margin in the remaining 62 (20.2%) patients.

**Table 2:** Baseline characteristics of pancreatic cancer patients who underwent upfront pancreaticoduodenectomy

Variables	Total *n* (%)	R0-wide *n* (%)	R0-narrow *n* (%)	R1 *n* (%)	*P* value
Number (%)	307 (100.0)	139 (45.3)	106 (34.5)	62 (20.2)	
Mean age; years (IQR)	67.0	67.0	67.5	70.0	0.223
	(60.0–74.0)	(59.0–72.0)	(61.0–74.0)	(59.3–75.8)	
Sex					0.283
Male	164 (53.4)	79 (56.8)	50 (47.2)	35 (56.5)	
Female	143 (46.6)	60 (43.2)	56 (52.8)	27 (43.5)	
ASA classification					0.598
I/II	270 (88.0)	125 (89.9)	92 (86.8)	53 (85.5)	
III/IV	37 (12.0)	14 (10.1)	14 (13.2)	9 (14.5)	
Resectability^a^					0.067
RPC	270 (88.0)	126 (90.6)	95 (89.6)	49 (79.0)	
BR/LAPC	37 (12.0)	13 (9.4)	11 (10.4)	13 (21.0)	
CA 19-9 (U/mL)^a^					0.359
>150	151 (49.2)	62 (44.6)	56 (52.8)	33 (53.2)	
≤150	156 (50.8)	77 (55.4)	50 (47.2)	29 (46.8)	
T stage					0.003
T1/T2	276 (89.9)	130 (93.5)	98 (92.5)	48 (77.4)	
T3/T4	31 (10.1)	9 (6.5)	8 (7.5)	14 (22.6)	
N stage					0.003
N0	85 (27.7)	52 (37.4)	20 (18.9)	13 (21.0)	
N1/N2	222 (72.3)	87 (62.6)	86 (81.1)	49 (79.0)	
Complication					0.770
Yes	88 (28.7)	37 (26.6)	32 (30.2)	19 (30.6)	
No	219 (71.3)	102 (73.4)	74 (69.8)	43 (69.4)	
Adjuvant treatment					0.254
No	61 (19.9)	26 (18.7)	19 (17.9)	16 (25.8)	
CTx.	147 (47.9)	66 (47.5)	58 (54.7)	23 (37.1)	
CCRT	99 (32.2)	47 (33.8)	29 (27.4)	23 (37.1)	

*IQR* interquartile range; *ASA* American Society of Anesthesiologists; *RPC* resectable pancreatic cancer; *BR/LAPC* borderline resectable/locally advanced pancreatic cancer; *CA 19-9* carbohydrate antigen 19-9; *CTx* chemotherapy; *CCRT* concurrent chemo-radiotherapy

^a^Description based on the time of diagnosis

Most characteristics did not differ significantly among the three groups stratified by resection margin status. However, the proportion of patients with borderline resectable or locally advanced PDAC was marginally significantly higher in the R1 (21.0%) group than in the R0-wide (9.4%) and R0-narrow (10.4%) groups (*P* = 0.067). An R1 margin also was associated with a higher pathologic T stage (*P* = 0.003). The proportion of patients with metastatic lymph nodes was significantly lower in the R0-wide group (62.6%) than in the R0-narrow (81.1%) and R1 (79.0%) (*P* = 0.003) groups.

The baseline characteristics of the 97 patients who underwent NAC followed by PD are summarized in Table 3. The median age was 60 years (IQR, 56–67 years) years. Of the 97 patients, 52 were men (53.6%), and 45 were women (46.4%). An R0-wide margin was achieved in 63 (64.9%) patients, an R0-narrow margin in 22 (22.7%) patients, and an R1 margin in the remaining 12 (12.4%) patients. Most characteristics did not differ significantly among the three groups stratified by resection margin status. However, the proportion of patients with borderline resectable or locally advanced PDAC was marginally significantly higher in the R1 (100.0%) group than in the R0-wide (69.8%) group (*P* = 0.063). Based on the pathologic T stage, the proportion of patients with stage T3 disease or worse was higher in the R1 (33.3%) group than in the R0-wide (9.5%) and R0-narrow (4.5%) groups (*P* = 0.044). Regarding pathologic N stage, the proportion of patients with metastatic lymph nodes was lower in the R0-wide (38.1%) group than in the R0-narrow (63.6%) and R1 (75.0%) groups (*P* = 0.017).

**Table 3:** Baseline characteristics of pancreatic cancer patients who underwent neoadjuvant chemotherapy followed by pancreaticoduodenectomy

Variables	Total *n* (%)	R0-wide *n* (%)	R0-narrow *n* (%)	R1 *n* (%)	*P* Value
Number (%)	97 (100.0)	63 (64.9)	22 (22.7)	12 (12.4)	
Median age: years (IQR)	60.0	62.0	57.5	60.0	0.139
	(56.0–67.0)	(56.0–69.0)	(55.0–64.0)	(57.0–62.8)	
Sex					0.787
Male	52 (53.6)	32 (50.8)	13 (59.1)	7 (58.3)	
Female	45 (46.4)	31 (49.2)	9 (40.9)	5 (41.7)	
ASA classification					0.607
I/II	81 (83.5)	51 (81.0)	20 (90.9)	10 (83.3)	
III/IV	16 (16.5)	12 (19.0)	2 (9.1)	2 (16.7)	
Resectability^a^					0.063
RPC	24 (24.7)	19 (30.2)	5 (22.7)	0 (0.0)	
BR/LAPC	73 (75.3)	44 (69.8)	17 (77.3)	12 (100.0)	
CA 19-9 (U/mL)^a^					0.195
>150	34 (35.1)	21 (33.3)	6 (27.3)	7 (58.3)	
≤150	63 (64.9)	42 (66.7)	16 (72.7)	5 (41.7)	
Response to NAC					0.505
CR/PR	33 (34.0)	24 (38.1)	5 (22.7)	4 (33.3)	
SD/PD	64 (66.0)	39 (61.9)	17 (77.3)	8 (66.7)	
T stage					0.044
T1/T2	86 (88.7)	57 (90.5)	21 (95.5)	8 (66.7)	
T3/T4	11 (11.3)	6 (9.5)	1 (4.5)	4 (33.3)	
N stage					0.017
N0	50 (51.5)	39 (61.9)	8 (36.4)	3 (25.0)	
N1/N2	47 (48.5)	24 (38.1)	14 (63.6)	9 (75.0)	
Complication					0.190
Yes	26 (26.8)	18 (28.6)	3 (13.6)	5 (41.7)	
No	71 (73.2)	45 (74.4)	19 (86.4)	7 (58.3)	
Perioperative RTx.					0.724
No	44 (45.4)	26 (41.3)	12 (54.6)	6 (50.0)	
Pre-SABR	16 (16.5)	12 (19.0)	3 (13.6)	1 (8.3)	
Pre-CCRT	18 (18.5)	11 (17.5)	3 (13.6)	4 (33.4)	
Post-CCRT	19 (19.6)	14 (22.2)	4 (18.2)	1 (8.3)	
Adjuvant CTx.					>0.99
No	7 (7.2)	5 (7.9)	1 (4.5)	1 (8.3)	
Yes	90 (92.8)	58 (92.1)	21 (95.5)	11 (91.7)	

*IQR* interquartile range; *ASA* American Society of Anesthesiologists; *RPC* resectable pancreatic cancer; *BR/LAPC* borderline resectable/locally advanced pancreatic cancer; *CA 19-9* carbohydrate antigen 19-9; *NAC* neoadjuvant chemotherapy; *CR* complete response; *PR* partial response; *SD* stable disease; *PD* progressive disease; *RTx* radiotherapy; *SABR* stereotactic ablative radiotherapy; *CCRT* concurrent chemo-radiotherapy; *CTx* chemotherapy

^a^Description based on the time of diagnosis

### Impact of Resection Margin Status on OS

**Fig. 1:**
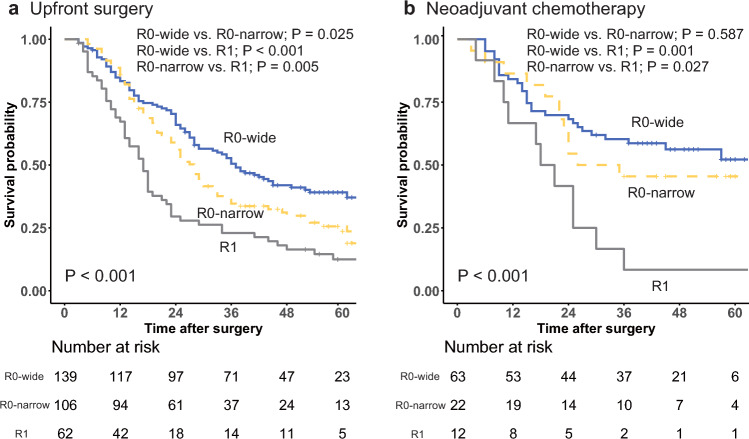
Overall survival according to resection margin status (**a**) in upfront pancreaticoduodenectomy settings, and (**b**) in neoadjuvant chemotherapy followed by pancreaticoduodenectomy settings

For the patients who underwent upfront PD, the KM curves for OS according to the resection margin status are shown in Fig. 1a. The patients with an R0-wide margin showed a significantly higher 5-year OS rate (39.1%; 95% CI, 31.6–48.5%) than those with an R0-narrow margin (25.6%; 95% CI, 18.0–36.3%; *P* = 0.025). In addition, the 5-year OS rate was significantly higher for the patients with an R0-narrow margin than for those with an R1 margin (12.5%; 95% CI, 6.3–24.7%; *P* = 0.005).

The KM curves for OS according to the resection margin status of the patients who underwent NAC followed by PD are shown in Fig. 1b. Unlike the patients who underwent upfront PD, the patients with an R0-wide margin had a 5-year OS rate (52.2%; 95% CI, 40.0–68.1%) that did not differ significantly from that for the patients with an R0-narrow margin (45.5%; 95% CI, 28.8–71.8%) (*P* = 0.587). However, similar to the upfront PD group, the patients with an R0-narrow margin showed a significantly higher 5-year OS rate (8.3%; 95% CI, 1.3–54.4%; *P* = 0.027) than those with an R1 margin.

In multivariate Cox regression analyses for OS, lymph node metastasis and R1 margin were associated with shorter OS for both the patients who underwent upfront PD and those who underwent NAC followed by PD (Table S1).

### Impact of Resection Margin Status on Recurrence

**Fig. 2:**
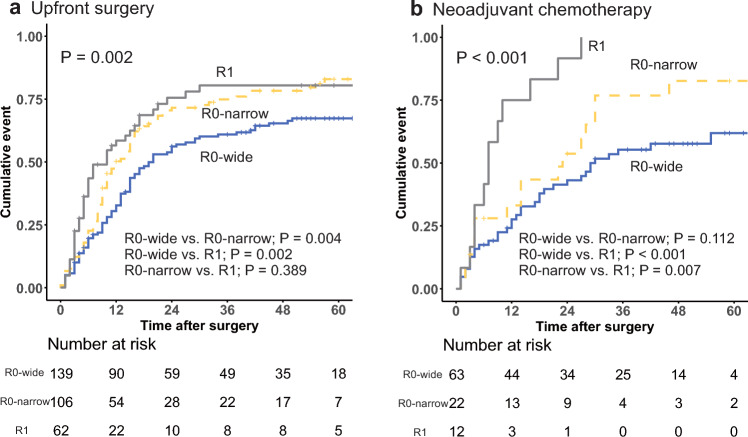
Time to overall recurrence from pancreaticoduodenectomy according to resection margin status (**a**) in upfront pancreaticoduodenectomy settings, and (**b**) in neoadjuvant chemotherapy followed by pancreaticoduodenectomy settings

The cumulative risk of overall recurrence according to resection margin status for the patients who underwent upfront PD is shown in Fig. 2a. The median time to overall recurrence was significantly longer for the patients who achieved an R0-wide margin (20.4 months) than for those with an R0-narrow margin (13.0 months; *P* = 0.004) and those with an R1 margin (10.1 months; *P* = 0.002). The median time until overall recurrence did not differ significantly between the patients with an R0-narrow margin and those with an R1 margin (*P* = 0.389).

To evaluate the direct effect of resection margin status on recurrence, uni- and multivariate Cox regression analyses of LRR were performed (Table 4). The univariate analyses identified high CA 19-9 (>150 U/mL), lymph node metastasis, resection margin status, and concurrent chemo-radiotherapy after surgery as significant predictors of LRR. In the multivariate analyses, the risk factors for LRR were high CA 19-9 (HR, 1.44; 95% CI, 1.03–2.02; *P* = 0.033), lymph node metastasis (HR, 1.96; 95% CI, 1.29–2.99; *P* = 0.002), and tumor-free margin smaller than 1 mm (HR, 1.37 [95% CI, 0.94–2.00; *P* = 0.098] for R0-wide vs R0-narrow and HR, 1.64 [95% CI, 1.02–2.65; *P* = 0.043] for R0-wide vs R1). Concurrent chemo-radiotherapy after surgery was associated with lower risk of LRR than that for the patients who did not undergo adjuvant treatment (HR, 0.45; 95% CI, 0.27–0.74; *P* = 0.002). However, only chemotherapy after surgery was not associated with the risk of LRR compared with that for the patients who did not undergo adjuvant treatment (HR, 0.88; 95% CI, 0.56–1.37; *P* = 0.565).

**Table 4:** Risk factors for locoregional recurrence in pancreatic cancer patients who underwent upfront pancreaticoduodenectomy

Variables	Univariate analysis		Multivariate analysis	
	HR (95% CI)	*P* value	HR (95% CI)	*P* value
Age (years)				
≤60 (*n* = 83)	1.00 (Reference)			
>60 (*n* = 224)	0.99 (0.68–1.43)	0.947		
Sex				
Male (*n* = 164)	1.00 (Reference)			
Female (*n* = 143)	0.79 (0.56–1.10)	0.165		
ASA classification				
I/II (*n* = 270)	1.00 (Reference)			
III/IV (*n* = 37)	1.09 (0.65–1.84)	0.735		
Resectability^a^				
RPC (*n* = 270)	1.00 (Reference)			
BR/LAPC (*n* = 37)	1.27 (0.77–2.08)	0.352		
CA 19-9 (U/mL)^a^				
≤150 (*n* = 151)	1.00 (Reference)		1.00 (Reference)	
>150 (*n* = 156)	1.51 (1.08–2.11)	0.015	1.44 (1.03–2.02)	0.033
T stage				
T1/T2 (*n* = 276)	1.00 (Reference)			
T3/T4 (*n* = 31)	1.29 (0.74–2.24)	0.374		
N stage				
N0 (*n* = 85)	1.00 (Reference)			
N1/N2 (*n* = 222)	2.13 (1.41–3.21)	< 0.001	1.96 (1.29–2.99)	0.002
R status				
R0-wide (*n* = 139)	1.00 (Reference)		1.00 (Reference)	
R0-narrow (*n* = 106)	1.67 (1.16–2.41)	0.006	1.37 (0.94–2.00)	0.098
R1 (*n* = 62)	1.66 (1.04–2.65)	0.035	1.64 (1.02–2.65)	0.043
Adjuvant treatment				
No (*n* = 61)	1.00 (Reference)		1.00 (Reference)	
CTx. (*n* = 147)	0.91 (0.59–1.41)	0.676	0.88 (0.56–1.37)	0.565
CCRT (*n* = 99)	0.48 (0.29–0.79)	0.004	0.45 (0.27–0.74)	0.002

*HR* hazard ratio; *CI* confidence interval; *ASA* American Society of Anesthesiologists; *RPC* resectable pancreatic cancer; *BR/LAPC* borderline resectable/locally advanced pancreatic cancer; *CA 19-9* carbohydrate antigen 19-9; *CTx* chemotherapy; *CCRT* concurrent chemo-radiotherapy

^a^Description based on the time of diagnosis

The cumulative risk of overall recurrence according to resection margin status for the patients who underwent NAC followed by PD is shown in Fig. 2b. The median time to overall recurrence was significantly shorter for the patients who underwent R1 resection (7.1 months) than for those with an R0-wide margin (29.5 months; P < 0.001) and those with an R0-narrow margin (23.8 months; P = 0.007). The median time until overall recurrence did not differ significantly between the patients with an R0-wide margin and those with an R0-narrow margin (*P* = 0.112).

Uni- and multivariate Cox regression analyses were performed for LRR (Table 5). The univariate analyses identified lymph node metastasis, resection margin status, and perioperative radiotherapy as significant predictors of LRR. In multivariate analyses, the independent risk factors for LRR were lymph node metastasis (HR, 2.09; 95% CI, 1.00–4.35; *P* = 0.049) and R1 resection margin (HR, 3.57 [95% CI, 1.41–9.09; *P* = 0.007] for R0-wide vs R1). Unlike the risk factors for the patients who underwent upfront PD, an R0-narrow margin was not identified as a significant risk factor for LRR compared with an R0-wide margin (HR, 1.29; 95% CI, 0.57–2.95; *P* = 0.543). Similar to the risk factors for the patients who underwent upfront PD, concurrent chemo-radiotherapy after surgery was associated with lower risk of LRR than that for the patients who did not undergo perioperative radiotherapy (HR, 0.27; 95% CI, 0.08–0.93; *P* = 0.038).

**Table 5:** Risk factors for locoregional recurrence in patients who underwent neoadjuvant chemotherapy followed by pancreaticoduodenectomy

Variables	Univariate analysis		Multivariate analysis	
	HR (95% CI)	*P* value	HR (95% CI)	*P* value
Age (years)				
≤60 (*n* = 49)	1.00 (Reference)			
>60 (*n* = 48)	0.67 (0.34–1.31)	0.238		
Sex				
Male (*n* = 52)	1.00 (Reference)			
Female (*n* = 45)	1.06 (0.55–2.04)	0.858		
ASA classification				
I/II (*n* = 81)	1.00 (Reference)			
III/IV (*n* = 16)	1.20 (0.52–2.74)	0.668		
Resectability^a^				
RPC (*n* = 24)	1.00 (Reference)			
BR/LAPC (*n* = 73)	1.58 (0.69–3.60)	0.281		
CA 19-9 (U/mL)^a^				
≤150 (*n* = 34)	1.00 (Reference)			
>150 (*n* = 63)	1.19 (0.61–2.33)	0.614		
Response to NAC				
CR/PR (*n* = 33)	1.00 (Reference)			
SD/PD (*n* = 64)	1.39 (0.67–2.88)	0.380		
T stage				
T1/T2 (*n* = 86)	1.00 (Reference)			
T3/T4 (*n* = 11)	0.88 (0.27–2.88)	0.837		
N stage				
N0 (*n* = 50)	1.00 (Reference)		1.00 (Reference)	
N1/N2 (*n* = 47)	2.59 (1.30–5.14)	0.007	2.09 (1.00–4.35)	0.049
R status				
R0-wide (*n* = 63)	1.00 (Reference)		1.00 (Reference)	
R0-narrow (*n* = 22)	1.96 (0.89–4.28)	0.093	1.29 (0.57–2.95)	0.543
R1 (*n* = 12)	6.56 (2.77–15.50)	< 0.001	3.57 (1.41–9.09)	0.007
Perioperative RTx.				
No (*n* = 44)	1.00 (Reference)		1.00 (Reference)	
Pre-SABR (*n* = 16)	0.30 (0.09–1.02)	0.053	0.39 (0.11–1.34)	0.135
Pre-CCRT (*n* = 18)	0.76 (0.33–1.79)	0.536	0.72 (0.31–1.69)	0.449
Post-CCRT (*n* = 19)	0.22 (0.07–0.75)	0.015	0.27 (0.08–0.93)	0.038
Adjuvant CTx.				
No (*n* = 7)	1.00 (Reference)			
Yes (*n* = 90)	0.83 (0.20–3.48)	0.803		

*HR* hazard ratio; *CI* confidence interval; *ASA* American Society of Anesthesiologists; *RPC* resectable pancreatic cancer; *BR/LAPC* borderline resectable/locally advanced pancreatic cancer; *CA 19-9* carbohydrate antigen 19-9; *NAC* neoadjuvant chemotherapy; *CR* complete response; *PR* partial response; *SD* stable disease; *PD* progressive disease; *RTx* radiotherapy; *SABR* stereotactic ablative radiotherapy; *CCRT* concurrent chemo-radiotherapy; *CTx* chemotherapy

^a^Description based on the time of diagnosis

### Details about Individual Margin

The individual margin status is shown in Fig. S2. Overall, the distribution of margin involvement frequency (number of patients with individual margin involvement/number of all patients) between the patients who underwent upfront PD and those who underwent NAC followed by PD was similar. In both groups, the SMV margin was the most frequently involved, followed by the SMA margin and circumferential surfaces. However, the patients who underwent NAC followed by PD generally showed lower margin involvement at most margins than those who underwent upfront PD.

To evaluate the prognostic power of individual margins, we broadly divided them into controllable margins that can be determined by surgeons (SMV, SMA, neck, and bile duct) and uncontrollable margins (anterior and posterior), which are regarded as free surfaces rather than dissection margins. According to a previous analysis about the prognostic power of margin status, the 1-mm rule was applied to the patients who underwent upfront PD, whereas the 0-mm rule was applied to those who underwent NAC followed by PD. Involvement of controllable margins was always a significant risk factor for shorter OS and LRRFS in both treatment settings, whereas involvement of uncontrollable margins was a risk factor for OS in the upfront PD setting and for LRRFS in the NAC setting (Table S2).

## Discussion

The definition and prognostic power of the resection margin status for patients with PDAC who undergo PD remain controversial. In this situation, a novel treatment strategy (NAC) has made interpreting the clinical significance of the margin status more difficult. This study provided evidence that the wide resection margin of 1 mm or larger was associated with improved prognosis for the patients with PDAC who underwent upfront PD. In contrast, a narrow resection margin larger than 0 mm was associated with improved prognosis for the patients with PDAC who underwent NAC followed by PD. These findings suggest that the clinical significance of margin status may vary depending on the treatment strategy.

Previous studies have shown inconsistent results regarding the effect of margin involvement on the prognosis of patients with PDAC who underwent PD. The study design and the absence of important variables considered in this study, such as the details of perioperative treatment, may be related to these variations. For patients who underwent upfront PD, a recent meta-analysis by Leonhardt et al.^30^ reported that a 1 mm clearance rule has prognostic relevance because PDAC exhibits a diffuse infiltrative growth pattern. However, Kishi et al.^38^ reported that a 0-mm clearance rule may also have prognostic value in multivariate analysis.

In addition, the prognostic significance of the resection margin status of patients who underwent NAC followed by PD still is the subject of a few investigations with mixed results. Maeda et al.^25^ (2020) reported that the presence of tumor cells directly at the margin was an independent predictor of OS in patients treated with NAC and PD. However, Schmocker et al.^26^ reported that margin involvement did not affect prognosis when NAC was received, therefore necessitating vigorous surgical attempts after extensive NAC. Our study demonstrated that obtaining a wide resection margin (≥ 1 mm) is appropriate for patients who underwent upfront PD, and that obtaining only a narrow resection margin (>0 mm) is appropriate for patients who underwent NAC followed by PD. These findings are consistent with those of previous reports on the prognostic impact of the 1-mm rule for patients who underwent upfront PD and the tendency for reduced prognostic power of resection margin status after NAC. This implies that systemic control is crucial for PDAC and implies that the extent and role of local treatment need to be readjusted according to the level of systemic control.

The role of perioperative radiotherapy in PDAC is still another ongoing issue. Although the European Organization for Research and Treatment of Cancer and the European Study Group for Pancreatic Cancer-1 trial failed to demonstrate the benefit of adjuvant radiotherapy, several retrospective studies have suggested the benefits of adjuvant radiotherapy.^39–43^ Takahashi et al.,^43^ using the National Cancer Database, reported that adjuvant chemoradiation improved survival outcomes for patients with margin involvement regardless of nodal status. However, data to support the addition of radiotherapy together with chemotherapy after surgery remain sparse, and the significance of radiotherapy for patients who underwent NAC remains unclear.

In this study, we found that lymph node metastasis and margin involvement were significantly associated with shorter LRRFS and that concurrent chemoradiotherapy after surgery was significantly associated with longer LRRFS in both upfront PD and NAC followed by PD. Therefore, we believe that adding radiotherapy to chemotherapy after surgery is helpful for patients at high risk of LRR, such as those with positive margins or lymph node metastasis. Although we have discussed the possible benefits of adjuvant radiotherapy for patients with positive margins, randomized controlled trials are necessary to identify individuals who would benefit the most from perioperative radiotherapy.

Despite several efforts, the dilemmas faced by surgeons and pathologists in obtaining microscopically negative margins and defining positive margins persist. One of the issues is whether the anterior and posterior circumferential surfaces, which are regarded as free surfaces rather than dissection margins, should be taken into account when the margin status is assessed.^44^ Although the College of American Pathologists does not view the anterior circumferential surface as a true margin, the Royal College of Pathologists strongly advises reporting it due to an association of involvement of an anterior surface with LRR.^14,16^ In other gastrointestinal cancers such as stomach and colon cancer, tumor extension into the surrounding peritoneum is recorded in the T category of tumor-node-metastasis (TMN) stage rather than margin status.

The current study demonstrated the frequent involvement of the anterior and posterior circumferential surfaces in both upfront PD and NAC followed by PD. We also found that margins determined by surgeons always showed a significant impact on OS and LRRFS in any treatment strategy, whereas the anterior and posterior circumferential surfaces were identified as predictive factors for OS and LRRFS in specific treatment settings. Therefore, pathologists should constantly consider the significance of the anterior and posterior circumferential surfaces while reporting pathologic results, and surgeons should consider these points while assessing margins in the perioperative period. Because the results were similar in analysis only with controllable margins, surgeons should be careful when determining the extent of resection during surgery.

We acknowledge that this study had a few limitations. First, the major limitation of this study was its retrospective nature, which would be associated with selection bias and confounding factors. Pathologic characteristics other than stage and resection margin status may have been confounding factors, even if we covered a lot of clinical parameters.

Second, the fact that all the patients came from high-volume, specialized facilities could limit the broad application of these findings. In addition, the lack of international consensus for PD specimen-handling techniques and resection margin evaluation methods makes it difficult to generalize the findings of this study.

Third, the association between NAC, margin involvement, and survival may be muddled by the fact that one third of the patients received neoadjuvant radiotherapy. Conversely, this implies that the association between perioperative radiotherapy and locoregional failure may be muddled by combining with chemotherapy and resection margin status.

Fourth, because the use of NAC had just recently begun, despite the multicenter nature of this study, the number of patients who underwent NAC was rather limited. Therefore, subgroup analyses were inevitably limited, and their statistical power also was diminished, particularly for the patients who underwent NAC. Therefore, to assess the prognostic power of the resection margins in each subgroup according to the different perioperative treatment methods, including chemotherapy and radiotherapy, studies including more patients are required in the future.

## Conclusion

In summary, our results showed that obtaining a wide resection margin (≥1 mm) could enhance the prognosis of patients who underwent upfront PD, and that obtaining only a narrow resection margin (>0 mm) could be appropriate for patients who underwent NAC followed by PD. These findings suggest that the interpretation of margin status differs depending on the treatment strategy. Additionally, the potential benefits of adjuvant radiotherapy should be considered, especially for patients with margin involvement or metastatic lymph nodes.

## Supplementary Information

Below is the link to the electronic supplementary material.

Supplementary file1 (DOCX 653 kb)

## References

[1] Siegel RL, Miller KD, Wagle NS, Jemal A. Cancer statistics, 2023. *CA Cancer J Clin*. 2023;73:17–48. 36633525 10.3322/caac.21763

[2] Von Hoff DD, Ervin T, Arena FP, et al. Increased survival in pancreatic cancer with nab-paclitaxel plus gemcitabine. *N Engl J Med*. 2013;369:1691–703. 24131140 10.1056/NEJMoa1304369 PMC4631139

[3] Conroy T, Hammel P, Hebbar M, et al. FOLFIRINOX or gemcitabine as adjuvant therapy for pancreatic cancer. *N Engl J Med*. 2018;379:2395–406. 30575490 10.1056/NEJMoa1809775

[4] Tempero MA, Pelzer U, O’Reilly EM, et al. Adjuvant nab-paclitaxel + gemcitabine in resected pancreatic ductal adenocarcinoma: results from a randomized, open-label, phase III trial. *J Clin Oncol*. 2023;41:2007–19. 36521097 10.1200/JCO.22.01134 PMC10082313

[5] Park W, Chawla A, O’Reilly EM. Pancreatic cancer: a review. *JAMA*. 2021;326:851–62. 34547082 10.1001/jama.2021.13027 PMC9363152

[6] Verbeke CS, Leitch D, Menon KV, McMahon MJ, Guillou PJ, Anthoney A. Redefining the R1 resection in pancreatic cancer. *Br J Surg*. 2006;93:1232–7. 16804874 10.1002/bjs.5397

[7] Esposito I, Kleeff J, Bergmann F, et al. Most pancreatic cancer resections are R1 resections. *Ann Surg Oncol*. 2008;15:1651–60. 18351300 10.1245/s10434-008-9839-8

[8] Willett CG, Lewandrowski K, Warshaw AL, Efird J, Compton CC. Resection margins in carcinoma of the head of the pancreas: implications for radiation therapy. *Ann Surg*. 1993;217:144–8. 8094952 10.1097/00000658-199302000-00008 PMC1242753

[9] Nitecki SS, Sarr MG, Colby TV, van Heerden JA. Long-term survival after resection for ductal adenocarcinoma of the pancreas. Is it really improving? *Ann Surg*. 1995;221:59–66. 7826162 10.1097/00000658-199501000-00007 PMC1234495

[10] Sperti C, Pasquali C, Piccoli A, Pedrazzoli S. Survival after resection for ductal adenocarcinoma of the pancreas. *Br J Surg*. 1996;83:625–31. 8689203 10.1002/bjs.1800830512

[11] Millikan KW, Deziel DJ, Silverstein JC, et al. Prognostic factors associated with resectable adenocarcinoma of the head of the pancreas. *Am Surg*. 1999;65:618–23. 10399969

[12] Sohn TA, Yeo CJ, Cameron JL, et al. Resected adenocarcinoma of the pancreas-616 patients: results, outcomes, and prognostic indicators. *J Gastrointest Surg*. 2000;4:567–79. 11307091 10.1016/s1091-255x(00)80105-5

[13] Sohn HJ, Kim H, Kim SJ, et al. Oncologic outcomes according to the location and status of resection margin in pancreas head cancer: role of radiation therapy in R1 resection. *Ann Surg Treat Res*. 2022;102:10–9. 35071115 10.4174/astr.2022.102.1.10 PMC8753382

[14] Campbell F, Bennett M, Foulis A. Minimum dataset for histopathological reporting of pancreatic, ampulla of vater, and bile duct carcinoma. London: Royal College of Pathologists; 2002.

[15] Bockhorn M, Uzunoglu FG, Adham M, et al. Borderline resectable pancreatic cancer: a consensus statement by the international study group of pancreatic surgery (ISGPS). *Surgery*. 2014;155:977–88. 24856119 10.1016/j.surg.2014.02.001

[16] Washington K, Berlin J, Branton P, et al. Protocol for the examination of specimens from patients with carcinoma of the pancreas. *Coll Am Pathol.* 2016

[17] Ghaneh P, Kleeff J, Halloran CM, et al. The impact of positive resection margins on survival and recurrence following resection and adjuvant chemotherapy for pancreatic ductal adenocarcinoma. *Ann Surg*. 2019;269:520–9. 29068800 10.1097/SLA.0000000000002557

[18] You Y, Choi DW, Heo JS, et al. Clinical significance of revised microscopic positive resection margin status in ductal adenocarcinoma of pancreatic head. *Ann Surg Treat Res*. 2019;96:19–26. 30603630 10.4174/astr.2019.96.1.19 PMC6306502

[19] Tummers WS, Groen JV, Sibinga Mulder BG, et al. Impact of resection margin status on recurrence and survival in pancreatic cancer surgery. *Br J Surg*. 2019;106:1055–65. 30883699 10.1002/bjs.11115 PMC6617755

[20] Serenari M, Ercolani G, Cucchetti A, et al. The impact of extent of pancreatic and venous resection on survival for patients with pancreatic cancer. *Hepatobiliary Pancreat Dis Int*. 2019;18:389–94. 31230959 10.1016/j.hbpd.2019.06.004

[21] Jang JY, Han Y, Lee H, et al. Oncological benefits of neoadjuvant chemoradiation with gemcitabine versus upfront surgery in patients with borderline resectable pancreatic cancer: a prospective, randomized, open-label, multicenter phase 2/3 trial. *Ann Surg*. 2018;268:215–22. 29462005 10.1097/SLA.0000000000002705

[22] Versteijne E, van Dam JL, Suker M, et al. Neoadjuvant chemoradiotherapy versus upfront surgery for resectable and borderline resectable pancreatic cancer: long-term results of the Dutch randomized PREOPANC trial. *J Clin Oncol*. 2022;40:1220–30. 35084987 10.1200/JCO.21.02233

[23] Seufferlein T, Uhl W, Kornmann M, et al. Perioperative or only adjuvant gemcitabine plus nab-paclitaxel for resectable pancreatic cancer (NEONAX)-a randomized phase II trial of the AIO pancreatic cancer group. *Ann Oncol*. 2023;34:91–100. 36209981 10.1016/j.annonc.2022.09.161

[24] Labori KJ, Lassen K, Hoem D, et al. Neoadjuvant chemotherapy versus surgery first for resectable pancreatic cancer (Norwegian Pancreatic Cancer Trial-1 (NorPACT-1)): study protocol for a national multicentre randomized controlled trial. *BMC Surg*. 2017;17:94. 28841916 10.1186/s12893-017-0291-1 PMC6389186

[25] Maeda S, Moore AM, Yohanathan L, et al. Impact of resection margin status on survival in pancreatic cancer patients after neoadjuvant treatment and pancreatoduodenectomy. *Surgery*. 2020;167:803–11. 31992444 10.1016/j.surg.2019.12.008

[26] Schmocker RK, Delitto D, Wright MJ, et al. Impact of margin status on survival in patients with pancreatic ductal adenocarcinoma receiving neoadjuvant chemotherapy. *J Am Coll Surg*. 2021;232:405–13. 33338577 10.1016/j.jamcollsurg.2020.11.018

[27] Liao WC, Chien KL, Lin YL, et al. Adjuvant treatments for resected pancreatic adenocarcinoma: a systematic review and network meta-analysis. *Lancet Oncol*. 2013;14:1095–103. 24035532 10.1016/S1470-2045(13)70388-7

[28] Rashid R, Sohrabi C, Kerwan A, et al. The STROCSS 2024 guideline: strengthening the reporting of cohort, cross-sectional and case-control studies in surgery. *Int J Surg*. 2024;110:3151. 38445501 10.1097/JS9.0000000000001268 PMC11175759

[29] Kim KS, Kwon J, Kim K, Chie EK. Impact of resection margin distance on survival of pancreatic cancer: a systematic review and meta-analysis. *Cancer Res Treat*. 2017;49:824–33. 27561314 10.4143/crt.2016.336 PMC5512376

[30] Leonhardt CS, Niesen W, Kalkum E, et al. Prognostic relevance of the revised R status definition in pancreatic cancer: meta-analysis. *BJS Open*. 2022;6:zrac010. 35301513 10.1093/bjsopen/zrac010 PMC8931487

[31] Holm MB, Verbeke CS. Prognostic impact of resection margin status on distal pancreatectomy for ductal adenocarcinoma. *Curr Oncol*. 2022;29:6551–63. 36135084 10.3390/curroncol29090515 PMC9498008

[32] Edition S, Edge S, Byrd D. AJCC cancer staging manual. *AJCC Cancer Staging Manual.* 2017.

[33] Tempero MA, Malafa MP, Al-Hawary M, et al. Pancreatic adenocarcinoma, version 2.2021, NCCN clinical practice guidelines in oncology. *J Natl Compr Canc Netw*. 2021;19:439–57. 33845462 10.6004/jnccn.2021.0017

[34] Clavien PA, Barkun J, de Oliveira ML, et al. The Clavien-Dindo classification of surgical complications: five-year experience. *Ann Surg*. 2009;250:187–96. 19638912 10.1097/SLA.0b013e3181b13ca2

[35] Lee M, Kang JS, Kim H, et al. Impact of conversion surgery on survival in locally advanced pancreatic cancer patients treated with FOLFIRINOX chemotherapy. *J Hepatobiliary Pancreat Sci*. 2023;30:111–21. 34581022 10.1002/jhbp.1050

[36] Jung HS, Kwon W, Yun WG, et al. Optimal timing of surgery after neoadjuvant treatment in borderline resectable pancreatic cancer. *J Hepatobiliary Pancreat Sci*. 2024;31:737–46. 39034526 10.1002/jhbp.12049

[37] Benjamini Y, Hochberg Y. Controlling the false discovery rate: a practical and powerful approach to multiple testing. *J Royal Stat Soc Series B Methodol*. 1995;57:289–300.

[38] Kishi Y, Nara S, Esaki M, Hiraoka N, Shimada K. Feasibility of resecting the portal vein only when necessary during pancreatoduodenectomy for pancreatic cancer. *BJS Open*. 2019;3:327–35. 31183449 10.1002/bjs5.50130 PMC6551409

[39] Neoptolemos JP, Dunn JA, Stocken DD, et al. Adjuvant chemoradiotherapy and chemotherapy in resectable pancreatic cancer: a randomised controlled trial. *Lancet*. 2001;358:1576–85. 11716884 10.1016/s0140-6736(01)06651-x

[40] Klinkenbijl JH, Jeekel J, Sahmoud T, et al. Adjuvant radiotherapy and 5-fluorouracil after curative resection of cancer of the pancreas and periampullary region: phase III trial of the EORTC gastrointestinal tract cancer cooperative group. *Ann Surg*. 1999;230:776–82. 10615932 10.1097/00000658-199912000-00006 PMC1420941

[41] Corsini MM, Miller RC, Haddock MG, et al. Adjuvant radiotherapy and chemotherapy for pancreatic carcinoma: the Mayo Clinic experience (1975–2005). *J Clin Oncol*. 2008;26:3511–6. 18640932 10.1200/JCO.2007.15.8782

[42] Butturini G, Stocken DD, Wente MN, et al. Influence of resection margins and treatment on survival in patients with pancreatic cancer: meta-analysis of randomized controlled trials. *Arch Surg*. 2008;143:75–83. 18209156 10.1001/archsurg.2007.17

[43] Takahashi C, Shridhar R, Huston J, Meredith K. Adjuvant therapy for margin positive pancreatic cancer: a propensity score-matched analysis. *Pancreatology*. 2022;22:396–400. 35304103 10.1016/j.pan.2022.03.008

[44] Soer E, Brosens L, van de Vijver M, et al. Dilemmas for the pathologist in the oncologic assessment of pancreatoduodenectomy specimens: an overview of different grossing approaches and the relevance of the histopathological characteristics in the oncologic assessment of pancreatoduodenectomy specimens. *Virchows Arch*. 2018;472:533–43. 29589102 10.1007/s00428-018-2321-5 PMC5924671

