# Supplementary material for: Changing Clinical Meaning of Resection Margin Status According to the Treatment Paradigm and the Potential Role of Perioperative Radiotherapy for Patients with Pancreatic Ductal Adenocarcinoma: An Updated Multicenter Retrospective Cohort Study
Source: Ann Surg Oncol. 2025 Apr 30;32(7):5110–21. doi: 10.1245/s10434-025-17389-4 (PMC12129876; doi:10.1245/s10434-025-17389-4)
Supplement: Supplementary file 1 — Supplementary file1 (DOCX 653 kb) [file 10434_2025_17389_MOESM1_ESM.docx]

**Supplementary Materials - Index**

| **Supplementary Figures and Tables** |  |
| --- | --- |
| Supplementary Figure 1 | page. 2 |
| Supplementary Figure 2 | page. 3 |
| Supplementary Table 1 | page. 4 |
| Supplementary Table 2 | page. 5 |
|  |  |
|  |  |
|  |  |

**Supplementary FIG. 1** Flow chart showing study cohort. *PD*, pancreaticoduodenectomy.


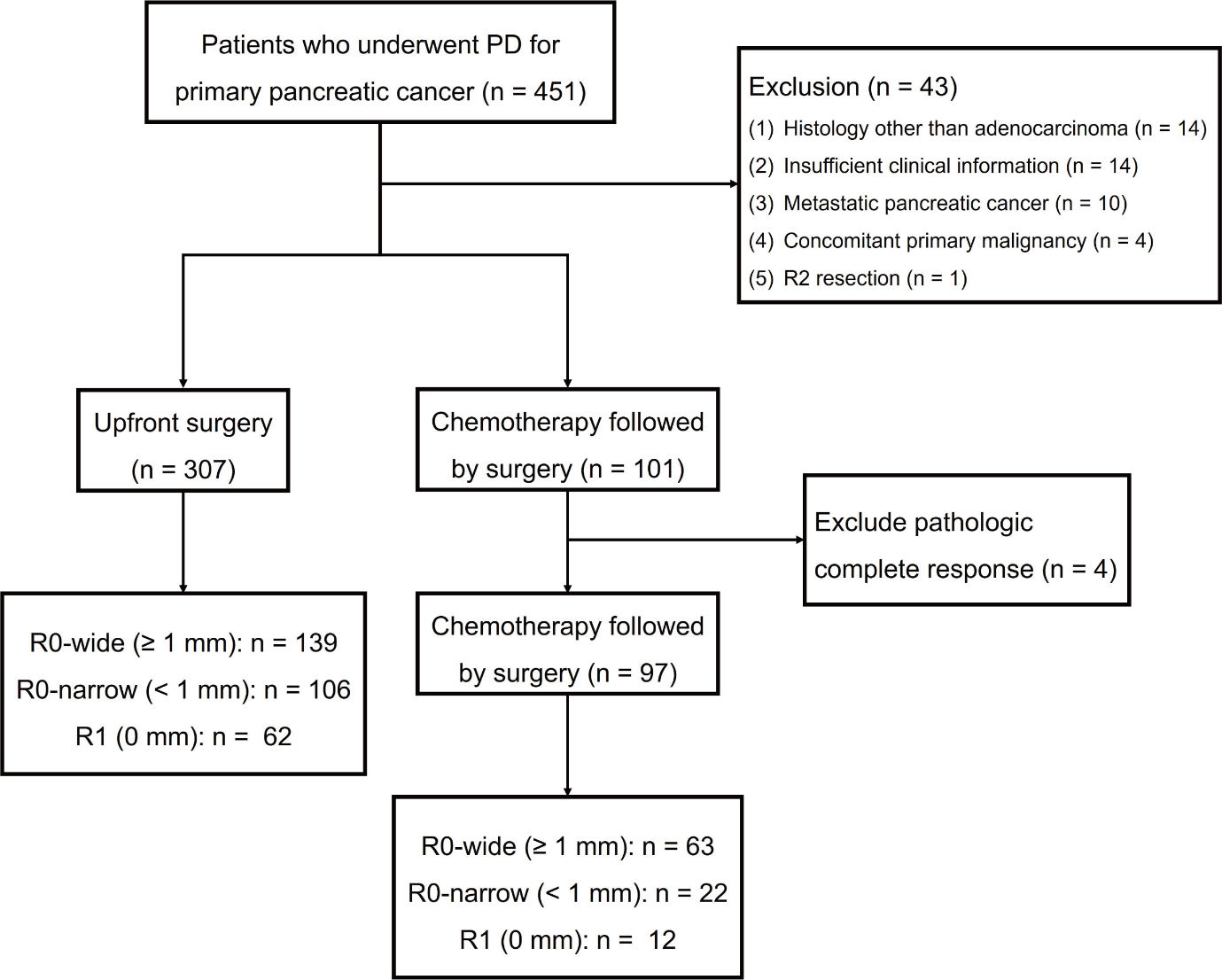


**Supplementary FIG. 2** Frequency of individual margin involvement according to the definition of positive margins in upfront pancreaticoduodenectomy settings **a**, and neoadjuvant chemotherapy followed by pancreaticoduodenectomy settings **b**. *SMV* superior mesenteric vein, *SMA* superior mesenteric artery


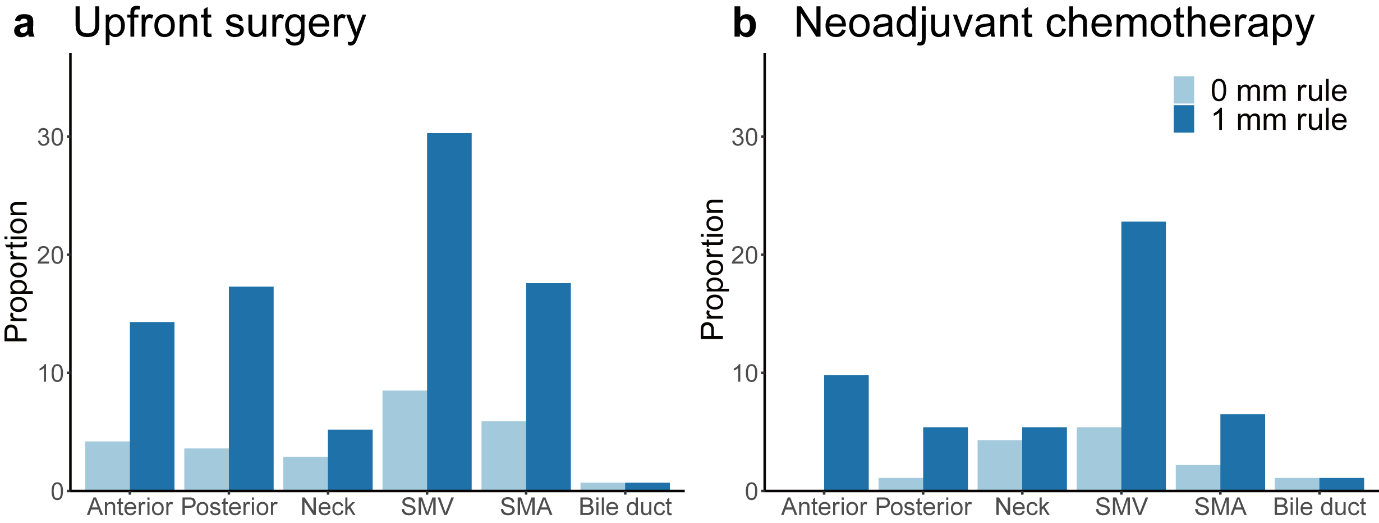


**Supplementary TABLE 1** Independent predictive factors for overall survival after resection in patients who underwent upfront pancreaticoduodenectomy and those who underwent neoadjuvant chemotherapy followed by pancreaticoduodenectomy, respectively.

| **Variables** | **Multivariate analysis** | |
| --- | --- | --- |
|  | **HR (95% CI)** | **P** |
| **Upfront surgery** |  |  |
| Resectability |  |  |
| RPC (N = 270) | 1.00 (Reference) |  |
| BR/LAPC (N = 37) | 1.19 (1.04, 2.23) | 0.031 |
| CA 19-9 (U/mL) |  |  |
| ≤ 150 (N = 151) | 1.00 (Reference) |  |
| > 150 (N = 156) | 1.54 (1.11, 2.13) | 0.010 |
| N stage |  |  |
| N0 (N = 85) | 1.00 (Reference) |  |
| N1/N2 (N = 222) | 2.28 (1.60, 3.26) | < 0.001 |
| R status |  |  |
| R0-wide (N = 139) | 1.00 (Reference) |  |
| R0-narrow (N = 106) | 1.17 (0.85, 1.61) | 0.333 |
| R1 (N = 62) | 1.92 (1.33, 2.77) | < 0.001 |
| Adjuvant treatment |  |  |
| N (N = 61) | 1.00 (Reference) |  |
| CTx. (N = 147) | 0.50 (0.36, 0.72) | < 0.001 |
| CCRT (N = 99) | 0.34 (0.23, 0.50) | < 0.001 |
| **Neoadjuvant chemotherapy** | | |
| N stage |  |  |
| N0 (N = 50) | 1.00 (Reference) |  |
| N1/N2 (N = 47) | 23.7 (1.32, 4.27) | 0.004 |
| R status |  |  |
| R0-wide (N = 63) | 1.00 (Reference) |  |
| R0-narrow (N = 22) | 1.04 (0.53, 2.05) | 0.910 |
| R1 (N = 12) | 2.48 (1.20, 5.09) | 0.014 |
| Adjuvant CTx. |  |  |
| N (N = 7) | 1.00 (Reference) |  |
| Y (N = 90) | 0.33 (0.13, 0.85) | 0.022 |

*HR* hazard ratio, *CI* confidence intervals, *RPC* resectable pancreatic cancer, *BR/LAPC* borderline resectable/locally advanced pancreatic cancer, *CA 19-9* carbohydrate antigen 19-9, *CTx* chemotherapy, *CCRT* concurrent chemo-radiotherapy

**Supplementary TABLE 2** Impact of margins determined by surgeons and circumferential surfaces on overall survival and locoregional recurrence-free survival

| **Variables** | **Overall survival** | | **Locoregional recurrence-free survival** | |
| --- | --- | --- | --- | --- |
|  | **HR (95% CI)** | **P** | **HR (95% CI)** | **P** |
| **Upfront surgery (1 mm)** |  |  |  |  |
| Controllable margins |  |  |  |  |
| R0 | 1.00 (Reference) |  | 1.00 (Reference) |  |
| R1 | 1.46 (1.12, 1.91) | 0.006 | 1.88 (1.35, 2.62) | <0.001 |
| Uncontrollable margins |  |  |  |  |
| R0 | 1.00 (Reference) |  | 1.00 (Reference) |  |
| R1 | 1.68 (1.26, 2.25) | <0.001 | 1.19 (0.82, 1.74) | 0.357 |
| **Neoadjuvant therapy (0 mm)** |  |  |  |  |
| Controllable margins |  |  |  |  |
| R0 | 1.00 (Reference) |  | 1.00 (Reference) |  |
| R1 | 2.74 (1.37, 5.50) | 0.005 | 4.77 (2.08, 10.94) | <0.001 |
| Uncontrollable margins |  |  |  |  |
| R0 | 1.00 (Reference) |  | 1.00 (Reference) |  |
| R1 | 2.67 (0.36, 19.57) | 0.334 | 6.98 (0.91, 53.45) | 0.061 |

*HR* hazard ratio, *CI* confidence intervals
